# Towards a Material-by-Design Approach to Electrospun Scaffolds for Tissue Engineering Based on Statistical Design of Experiments (DOE)

**DOI:** 10.3390/ma16041539

**Published:** 2023-02-12

**Authors:** Felicia Carotenuto, Noemi Fiaschini, Paolo Di Nardo, Antonio Rinaldi

**Affiliations:** 1 Dipartimento di Scienze Cliniche e Medicina Traslazionale, Università degli Studi di Roma “Tor Vergata”, Via Montpellier 1, 00133 Rome, Italy; 2 CIMER-Centro di Ricerca Interdipartimentale di Medicina Rigenerativa, Università degli Studi di Roma "Tor Vergata", Via Montpellier 1, 00133 Rome, Italy; 3 Nanofaber S.r.l., Via Anguillarese 301, 00123 Rome, Italy; 4 SSPT-PROMAS-MATPRO Laboratory, ENEA—Italian National Agency for New Technologies, Energy and Sustainable Economic Development, Via Anguillarese 301, 00123 Rome, Italy

**Keywords:** polycaprolactone, ECM, material genome initiative, rational design, biomaterials, electrospinning

## Abstract

Electrospinning bears great potential for the manufacturing of scaffolds for tissue engineering, consisting of a porous mesh of ultrafine fibers that effectively mimic the extracellular matrix (ECM) and aid in directing stem cell fate. However, for engineering purposes, there is a need to develop material-by-design approaches based on predictive models. In this methodological study, a rational methodology based on statistical design of experiments (DOE) is discussed in detail, yielding heuristic models that capture the linkage between process parameters (Xs) of the electrospinning and scaffold properties (Ys). Five scaffolds made of polycaprolactone are produced according to a 2^2^-factorial combinatorial scheme where two Xs, i.e., flow rate and applied voltage, are varied between two given levels plus a center point. The scaffolds were characterized to measure a set of properties (Ys), i.e., fiber diameter distribution, porosity, wettability, Young’s modulus, and cell adhesion on murine myoblast C1C12 cells. Simple engineering DOE models were obtained for all Ys. Each Y, for example, the biological response, can be used as a driver for the design process, using the process-property model of interest for accurate interpolation within the design domain, enabling a material-by-design strategy and speeding up the product development cycle. The implications are also illustrated in the context of the design of multilayer scaffolds with microstructural gradients and controlled properties of each layer. The possibility of obtaining statistical models correlating between diverse output properties of the scaffolds is highlighted. Noteworthy, the featured DOE approach can be potentially merged with artificial intelligence tools to manage complexity and it is applicable to several fields including 3D printing.

## 1. Introduction

Tissue Engineering and Regenerative Medicine (TERM) has been defined as a “rapidly growing interdisciplinary area which involves medical, biological, physical and engineering sciences to develop advanced strategies to repair, replace or regenerate tissues or organs damaged by traumatic events, disease or aging [1]. One of TERM’s main pursuits is the manufacture of tissues and organs obtained by combining biomaterials and cells [2,3]. In this field, biomaterials are used to create a scaffold emulating the extracellular matrix (ECM) of specific tissues. The scaffold provides appropriate biochemical and biophysical cues to support cell attachment, proliferation, and differentiation and promotes tissue regeneration and/or new tissue formation [4].

From a basic perspective, an engineered tissue may be represented as a compound of three key elements: (i) a scaffold that constitutes the mechanical and morphological backbone structure of the tissue, (ii) the cells that could be either differentiated tissue-specific ones or progenitor cells, and (iii) some specific biological and mechanical cues that would direct cell fate. These scaffolds are generally simpler than the ECM of organs and tissues and exhibit biocompatibility, tunable mechanical properties similar to those of the tissue to be replaced, and good porosity to facilitate cell infiltration [5,6,7,8]. In this paper, we focus on scaffold design and manufacturing using electrospinning, which is an engineering process that utilizes electrical forces to produce polymer fibers with diameters ranging from a few nm up to several micrometers from polymer solutions of both natural and synthetic polymers [9,10]. Electrospun scaffolds have been successfully used for a variety of TERM applications because they are biomimetic of the natural, fibrous ECM and contain a three-dimensional (3D) network of interconnected pores. [11]. Electrospun scaffolds can be endowed with a desired set of mechanical and chemical properties, as well as functional properties [11,12,13,14,15,16,17,18,19]. These scaffolds may be made from randomly oriented fibers, as well as aligned ones, with anisotropy in morphological and mechanical properties that can mimic the diverse ECM and provide an ideal substrate for specific cells to evolve into targeted tissues. For example, electrospun fiber alignment strongly affects myoblast differentiation, organization, and myotube formation [20,21,22]. 

Statistical design of experiments (DOE) can be used effectively towards the rationale design of a scaffold using electrospinning. A former study by our group [23,24] illustrates how DOE aids in establishing heuristic relationships between the process parameters (*Xs*) and the scaffold properties (*Ys*). Some of the most common *X* parameters are listed in Table 1, divided into three groups, namely: (i) materials and solution properties, (ii) processing conditions, and (iii) ambient conditions [7]. The effects of these factors have often been discussed in the literature with respect to the effects on the fiber diameter distribution, focusing on the correlation between fiber diameter and changes in materials and process parameters (i.e., polymer concentration, viscosity, conductivity, surface tension and dielectric constant of the polymer solution, flow (feed) rate of the polymer solution, nozzle diameter, electric field strength, and nozzle–substrate distance) [25,26,27,28,29]. 

So far, some effort has been devoted also to deriving mechanistic or physically based relationships for key parameters either of the process or of the resulting electrospun product, e.g., the critical extraction voltage marking the onset of electrospinning [30], the electrical field as a function of the spinneret [31], or the pore distribution in a randomly oriented fiber mat [32]. However, considering the intrinsic complexity of the electrospinning process, such modeling strategies are extremely complex and inherently limited in scope, whereas conversely, statistical methods, such as the DOE, are in comparison much simpler and more flexible and effective in mapping the electrospinning process for a given feedstock and application for engineering purposes. Furthermore, the DOE and regression methods are seemingly the best—if not the only—modeling option to correlate the biological response of a cell-engrafted scaffold vs. its microstructural and macroscopic properties. The possibility and the potential to deploy regression methods to interpret the biological response with regard just to a subset of scaffolds from the DOE was already discussed previously [24] in the context of the availability of incomplete or limited biological response data, which is often the case for large designs.

This paper is a follow-up study contemplating instead the case where the biological response is collected as one of the main responses of a DOE to investigate and optimize polycaprolactone (PCL) scaffolds for skeletal muscle tissue regeneration. Among synthetic polymers, PCL is a good test material for our study because it has been widely used for scaffold fabrication in the field of musculoskeletal system regeneration, being an FDA-approved polymer for health applications, and due to its high mechanical strength, good biocompatibility, slow degradation rate, and the less acidic breakdown of products in comparison to other polyesters [33]. A “2^k^ full-factorial” DOE was designed and implemented to quantify both the main and interaction effects of two selected process parameters, i.e., applied voltage and flow rate (*k* = 2), on the resulting nanofiber scaffold properties. The list of the chosen *Xs* and *Ys* in this study is summarized in Table 2 and Table 3, respectively.

Additionally, a “center point” is added to the basic full 2^2^ design to check for curvature effects. Table 4 reports the full experimental matrix displaying five treatments obtained for each combination of the Xs (expressed in coded variables) and the corresponding output Ys. 

As conveyed by Table 3 and Table 4, six fundamental descriptors are characterized and investigated using DOE: (i) the mean fiber diameter (FD), (ii) the FD spread, (iii) the porosity (*ε*%), (iv) the contact angle (CA), (v) the Young’s Modulus (E), and (vi) cell adhesion for a given cell line, which are referred as *Y*_1_, *Y*_2_, *Y*_3_, *Y*_4_, *Y*_5,_ and *Y*_6_ respectively. Generally, the biological performance depends on the optimal combination of all *Y*_1–5_ values for a given tissue type. However, determining such a combination is not trivial because each material parameter *Y_j_* (*j*:1…5) depends on a set of *N* controllable parameters (*X_i_*, *i*: 1…*N*) related to the process and raw materials that we express with an unknown functional (notation: lower capital *x* and *y* are actual values for parameters *X* and *Y*):

(1) $$y_{j}=f(x_{1},\ldots ,x_{N})$$

Many studies in the literature dissert about the specific effects of some *Xs* and *Y* outputs of interest for a given application, but they rarely actually focus on methodological aspects, nor estimate Equation (1) over a defined design domain, nor allow for a straight comparison between results from different authors. Determining a functional dependence *Y*(X) is a technological goal of consequence for enabling material-by-design manufacturing to direct function, architecture, scalability, and tailorability of a personalized scaffold, and ultimately develop the tissue engineering industry. 

Extensive parametric studies that can provide predictive frameworks for the electrospinning of fibers of the desired diameter range are in fact still uncommon in the literature, despite the potential industrial impact. As discussed here, parametric studies are one route to obtain the specific predictive tool aiding in the design of optimized fiber mats for applications closer to clinical applications in cardiac and musculoskeletal tissue engineering.

The objective of this study is to show how a controllable electrospinning process, designed and modeled using statistical DOE techniques, can foster the engineering of a complex multi-compartment and layered architecture scaffold, in principle optimized for many and diverse targeted cell types. As a proof of concept, we here propose a design of an electrospun PCL scaffold suitable for skeletal muscle tissue regeneration using C2C12 myoblasts as a cell model for illustrative purposes.

## 2. Materials and Methods

### 2.1. Factorial Design of Experiments (DOE)

To produce linear models approximating the relationships between parameters, such as Equation (1), and investigating the correlations among Xs and Ys, a “2^k^ factorial” design of experiments (DOE) (e.g., [34,35,36]), with k = 2 in our case (ref Table 2), was used. The fundamentals of the DOE theory relevant to our approach are recalled below including practical procedures to fit linear models using any modern statistical packages such as JMP-pro JMP-pro (SAS Institute, Cary, NC, USA) or MINITAB © (Minitab Inc., State College, PA, USA). 

The factorial DOE is a combinatorial strategy that provides an optimized strategy to obtain a first-order approximation of the model equation in Equation (1), capturing the dependence of each output variable Y_1–6_ in Table 3 on the Xs in Table 2. One distinctive feature of all DOE methods is the data collection using a rigidly planned, well-defined scheme. In our case, a total of five types of PCL scaffolds, corresponding to unique combinations of Xs levels and called “treatments”, were produced by considering all four possible combinations of the two levels of the two parameters in Table 2, plus a center point for the average level of all factors. All treatments were characterized experimentally to gather data to be analyzed with analysis of variance (ANOVA). Engaging in the collection and characterization of such a potentially large pre-determined dataset is motivated by key benefits including an increased accuracy of statistical estimates and confidence intervals, as well as orthogonality of the regression analysis, which are crucial for a systematic and comprehensive mapping of the electrospinning [23,24].

In our specific case, for any given Y output, the following linear model is fitted to the experimental data to obtain an “ordinary least square” linear estimate for *Y*(*X*_1_,*X*_2_):

*y* = *C*_0_ + *C*_*i*_*x*_*i*_ + *C*_12_*x*_1_*x*_2_     (i = 1,2) (2)

that encompasses four coefficient terms, two “main effects” for {*C*_1_,*C*_2_} and one “interaction” up to order 2 for {*C*_1_,*C*_2_,*C*_12_}. However, DOE models are better fitted to coded variables x* obtained from the natural variables using linear transformations:

(3) $$x_{i}^{*}(x_{i})=\frac{xi-\overset{-}{x}i}{(xHIGH-xLOW)/2}\;\;\;\;(i=1,2)$$

with HIGH and LOW mapping to +1 and −1 levels in Table 4, respectively, with Equation (2) rewritten as: 

*y* = *C*_0_*+*C*_i_*x_i_* + *C*_12_*x_1_*x_2_*     (i = 1,2) (4)

The choice of coded variables is implied throughout the DOE discussion hereafter, although the “*” superscripts will be dropped for readability’s sake. Table 4 renders a summary of the five treatments in coded form along with the mean responses for Y_1–6_, which allow grasping the marked variations between treatments at a glance. The actual order of the treatments from Table 4 was “randomized” during the execution of the experiment to mitigate the effect of ambient variables in Table 1, as customarily performed in the DOE [35]. 

Two of the crucial advantages of using coded variables are:

- The role of each parameter is normalized so that ranking {C_1_,C_2_,C_12_} immediately renders the relative importance of each parameter;
- The design is orthogonal so that each of the parameters {C_1_,C_2_,C_12_} can be estimated separately and dropped if not significant.

The latter condition means that the full model in Equation (4) may contain effect or interaction terms that are negligible in terms of statistical significance if the corresponding “*p*-value” statistics (i.e., Pearson value, herein defined as the probability of observing a certain value by chance against a reference statistical distribution) is higher than a threshold value, i.e., the significance level, chosen by the analysts based on the specific goal of the statistical study and nature of the dataset. A significance level of 10% is used throughout the discussion here, as it renders an appropriate sensitivity in our analysis to discriminate between significant and non-significant terms in Equation (4) fitted to our data. For more details about *p*-value and “hypothesis testing”, the reader is redirected to [34,35,36]. 

To assess the quality of the resulting DOE model from the ANOVA, the “coefficient of determination” *R*^2^ is the first indicator for the quality of fit and represents the percent of the variation in Y that is explained by the regression model. Being routinely computed by any commercial software, it increases from 0% to 100% as fit improves. Because the *R*^2^ monotonically increases with the number of parameters *p* in the model (excluding the constant term), it is often appropriate to consider also the *R*^2^-*adj* that is always less than *R*^2^ and is defined as:

(5) $$R_{adj}^{2}=1-(1-R_{}^{2})\frac{p}{n-p-1}$$

which adjusts the *R*^2^ for the number of predictors (*p*) relative to the number of data points (*n*) in multiple regression models encompassing two or more terms. The interested reader can reproduce the model results presented in this work for all Ys (ref. Table 5) by implementing a “backward-elimination” search algorithm to determine the optimal regression model, which was used systematically here in all cases and is available in many commercial statistical software programs (e.g., MINITAB, SAS, JMP-pro, etc.). 

### 2.2. Electrospinning Process and Solution Properties

A PCL solution was prepared from medical grade PCL granules (CAPA^®^ 6500, average 50,000 MW, Perstorp, Sweden) dissolved at 12% *w*/*v* concentration in a 65:35 solvent mixture of Chloroform (99.2% purity, stabilized with 0.6% ethanol, VWR, Radnor, PA, USA) and Dimethylformamide (100% purity, VWR, Radnor, PA, USA) under mild stirring overnight at room temperature. The supplier reported a theoretical density for PCL of ρ_0_ = 1.145 g/cm^3^. Non-woven scaffolds were fabricated with electrospinning using a pilot scale electrospinning station (Fluidnatek LE100, Bioinicia, Spain) using different variations of a process recipe, kindly provided by the Company Nanofaber srl (Rome, Italy) as indicated in Table 4, and varying the applied voltage and flow rate (FR).

### 2.3. Morphological Characterization and Fiber Distribution Estimate

**Figure 1:**
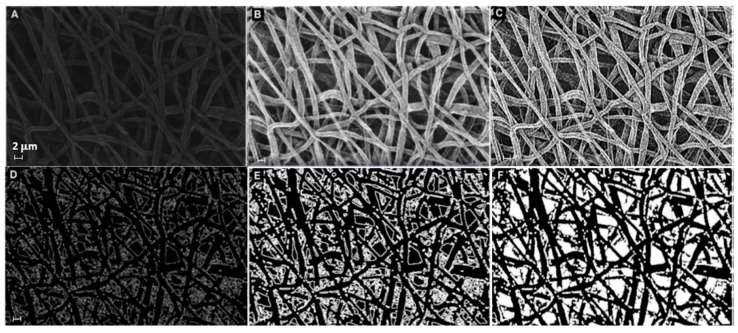
Sequence of image processing using MATLAB functions to prepare the input file for DiameterJ: (**A**) original SEM, (**B**) applied “histeq” and “medfilt2” functions, (**C**) applied “imsharpen”, “smoothing”, and “cleaning” functions, (**D**) applied “find edge” function, (**E**) applied “imdilate” function, (**F**) performed “imfill” function using ImageJ (scalebar is the same and equal 2 µm for all panels).

**Figure 2:**
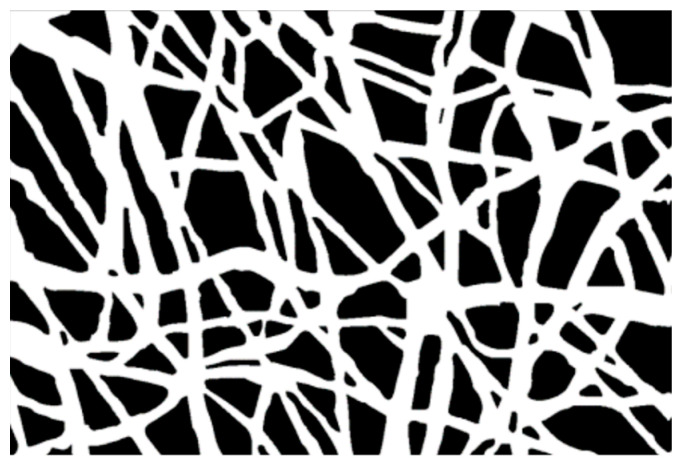
The image after ‘image trace’ operation (in Adobe Illustrator).

The microstructural texture of the electrospun mats was examined using field emission gun scanning electron microscopy (Leo 1530, ZEISS, Germany) operated at low voltage (i.e., 1–5 kV range) to investigate the fiber diameter without metal coating. The membrane thickness was also evaluated using SEM on the sample cross-section. Micrograph images at 5× magnification were taken and saved to be used in the analysis of fiber diameter, by sampling at least 30 individual fibers, with the software "ImageJ" (Rasband, W.S., ImageJ, U. S. National Institutes of Health, Bethesda, MD, USA, http://imagej.nih.gov/ij/, accessed on 30 December 2022, 1997–2022). The mean value and root mean square error (RMS) of the FD sample distribution were computed for each treatment over three micrographs taken randomly at different locations. Each image was segmented, i.e., transformed into binary images with white fibers on black background, and then analyzed after extracting a sample distribution of fiber diameters using DiameterJ [37]. As additional pre-processing, the segmentation was elaborated beforehand using MATLAB software (The MathWorks, Natick, MA, USA), applying the sequence functions in Figure 1. Image histogram equalization was operated on the starting SEM micrograph (Figure 1A) to increase the contrast, reduce noise, and preserve the structure edges. Moreover, local thresholding was performed to separate the outer fiber network from the background. The sequence of morphological operations, in the same way as described in the first strategy, followed. Finally, the filling function was performed manually on the latter binary image using “Image J” software. The result of this sequence of image processing steps is illustrated in the example in Figure 1, which is a black and white image (Figure 1F) that can be further treated in order to make the segmented object look more natural and smooth, e.g., using Adobe Illustrator (Adobe Illustrator, Adobe Inc., San Jose, CA, USA), such as in Figure 2. 

The output parameters *Y*_1_ and *Y*_2_ in Table 4 for each treatment were obtained as the grand mean and the pooled RMS FD from three samples, each with a number of observations *n_i_*

(6) $$RMS_{p}={\left(\frac{\sum_{i=1}^{3}\left(n_{i}-1\right)RMS_{i}^{2}}{\sum_{i=1}^{3}\left(n_{i}-1\right)}\right)}^{1/2}$$

These two output parameters provide a simple and sufficient description of the fiber distribution in terms of the expected value and spread, whereas higher-order descriptors such as kurtosis and skewness are out of scope. 

### 2.4. Porosity

The porosity of the electrospun scaffolds was evaluated [7] by weighing rectangular cut-out samples on a calibrated scale (ORMA, BCA120, UK) of known volume (Vol), such that the apparent density of the scaffolds ρ∗ is calculated using the following formula

ρ∗ = Mass(*g*)/Vol (cm^3^) (7)

and the percent scaffold porosity is obtained as

*ε*% = (1 − ρ∗/ρ_0_ × 100 (8)

where ρ_0_ = 1.145 g/cm^3^. Porosity measurements were performed in triplicate for each scaffold.

### 2.5. Statistical Contact Angle

The water contact angles of the PCL electrospun samples were measured using an optical contact angle measuring system, OCA 20 (DataPhysics, Germany), equipped with SCA 202 software, using ultra-pure water and operating at room temperature. Determinations were made using the sessile drop method with 10 μL droplet volume and a deposition rate of 10 µL/10 s in compliance to the European Standard UNI EN 15802 [38]. Ten measurements were carried out for each sample from different locations, and the average value is reported with its standard deviation (SD). The static contact angle *ϑ*, in degrees, is estimated from the diameter of the contact surface (*d*) and the height of the drop (*h*) as:

*ϑ* = 2 *arc*tg(2*h*/*d*) (9)

### 2.6. Mechanical Characterization

The tensile properties were evaluated using a micro-tensile loading frame (MICROTEST 200, DEBEN, UK) equipped with a 200 N load cell. The scaffolds under consideration represent randomly homogeneous materials with an in-plane isotropic linear-elastic behavior conveyed by Young’s Modulus. Rectangular specimens with a length of 20 mm and a width of 5 mm were cut from the electrospun sheets and submitted to quasi-static tensile testing at a strain rate of $\dot{\varepsilon }=0.005$. Scaffold thickness varied from sample to sample and was measured using SEM, rendering an average thickness of 40 μm for T1, 53 μm for T2, 62 μm for T3, 57 μm for T4, and 64 μm for T5 (with accuracy within ±10%). Stresses were computed by dividing the measured force by the apparent cross-sectional area, whereas strain was calculated from the cross-head displacement divided by the initial gage length at rest. Young’s modulus *E* was estimated as the slope of the linear fit to the initial portion of each stress–strain curve (with *R*^2^ > 99% in all cases, ref. Supplementary Material). For each sample type, the experiment was performed in triplicate, and the mechanical output parameter *Y*_5_ was valorized to the mean representative value.

### 2.7. Biological Evaluation 

To verify the biocompatibility of the scaffolds, a murine myoblast C2C12 cell line was utilized. Cells were cultured in Dulbecco’s modified Eagle medium (DMEM, Gibco) containing 15% fetal bovine serum (FBS, Gibco), L-glutamine 2 mM, and gentamicin 50 μg/ml (Sigma Aldrich, Inc., St. Louis, MO, USA). Before cell seeding, disks were cut out for each scaffold to fit tightly into 24-multiwell plates. The scaffolds were soaked in 90% ethanol solution for 30 min, then dried in a sterile ventilated biological hood and irradiated with UV light for 15 min. Subsequently, the scaffolds were washed with sterile phosphate-buffered saline (PBS) and equilibrated with DMEM containing 20% FBS for 24 h at 37 °C and 5% CO_2_ [39] to ensure the scaffolds was fully wet. A total of 4 × 10^4^ C2C12 cells was seeded for each scaffold using 24-well plates. The C2C12 cells cultured into wells without scaffolds were considered as the control group. After 48 h of culture, the cells were detached using a solution containing trypsin 0.05% in EDTA (Sigma Aldrich) and counted using a Bürker chamber after Trypan blue staining. Each value was normalized by cell number adhering on the bottom of the corresponding well and taken as the cell adhesion output parameter *Y*_6_, taken here as an indicator of the biocompatibility of a scaffold. After 4 days of cell culture, the cell morphology and cell viability were investigated on the selected scaffolds using immunofluorescence analysis. The cells seeded on scaffolds were fixed with ice-cold methanol (−20 °C) for 10 min. After saturation with Bovine serum albumin (BSA) 3%, the cells were incubated with tetra-rhodamine-conjugated phalloidin, directed against F-actin (Invitrogen Corp, Waltham, MA, USA) for 1 h at room temperature. The nuclei were stained with 4’,6’-diamidino-2-phenylindole (DAPI; Sigma-Aldrich). The images were taken using a Leica DMRB microscope (Wetzlar, Germany) equipped with a digital camera. Experiments were repeated 3 times. Data are expressed as the mean ± SD. Statistical analyses were performed with GraphPad Prism Software (GraphPad Software, San Diego, CA, USA). Differences were analyzed using a one-way analysis of variance (ANOVA). *p*-values < 0.05 were considered to indicate significance.

## 3. Results

### 3.1. Scaffold Characterization Results 

The results of the characterization of the five electrospun scaffolds are reported in Table 4 and show evidence of significant changes in scaffold properties across the treatments. While it is generally reported, for example, that smaller diameter fibers are obtained by either decreasing polymer concentration in the solution, decreasing the solution viscosity, increasing the solution conductivity, or decreasing surface tension and increasing the field strength (e.g., [25,40,41]), our analysis clearly highlights that the effects of interactions terms can deeply change the main effects of single parameters and can indeed revert such trends between the process parameters and scaffolds properties. After examining the scaffold properties, a statistical analysis is presented in Section 3.2 to support this statement.

#### 3.1.1. Fiber Diameter Distribution: Y_1_ and Y_2_

**Figure 3:**
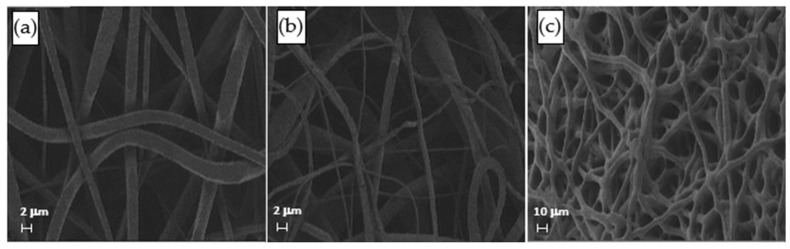
SEM micrographs of the samples (**a**) T1, (**b**) T2, and (**c**) T4.

**Figure 4:**
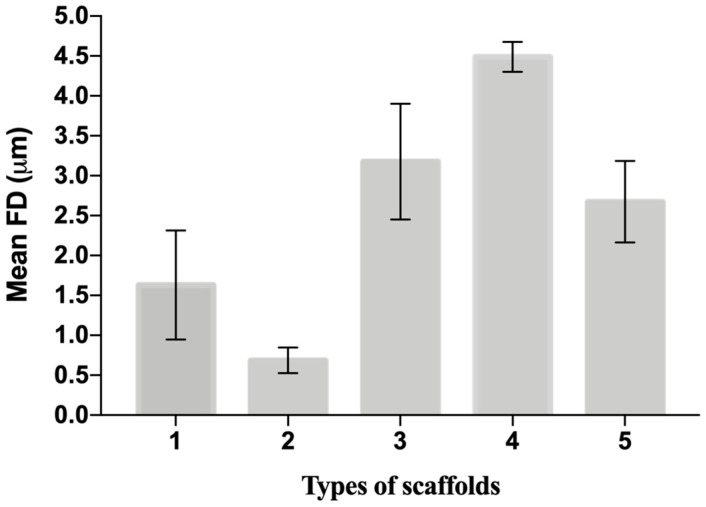
The mean value and spread of the first five scaffolds from DiameterJ. The histogram shows simultaneously the *Y*_1_ and *Y*_2_ of each scaffold from Table 4. Data are expressed as the mean ± SD (*p*-values < 0.05 is the significance interval).

The distribution of fiber diameters (FDs) is nearly universally regarded and investigated as the prime property to steer electrospun scaffold performance in terms of cell response. Common electrospun scaffolds usually exhibit a unimodal statistical distribution, the mean and spread of which represent the two minimal statistical descriptors of microscale architecture (i.e., *Y*_1_ and *Y*_2_ here) and can be tweaked using the process parameters. The different scaffold types exhibit very distinctive microstructures as revealed using SEM (Figure 3), which underlines the broadly different estimates of *Y*_1_ from DiameterJ ©, as displayed in Figure 4 at a glance. The largest and smallest fiber diameters were obtained for treatment T4 (4.49 μm) and T2 (0.69 μm). Noteworthy, at the given value of the FR, a decrease in fiber diameter for higher V is observed by comparing the pair T1–T2 (@*FR* = −1) vs. the pair T3–T4 (@*FR* = +1), in agreement with the literature [42,43]. As discussed later, though, the interplay (interaction) between the *FR* and *V* is crucial to determining the large differences in the FD spread, ranging from hundreds of nanometers for T2-T4 to above 1 micrometer for T1–T3. 

#### 3.1.2. Porosity, Wettability, and Mechanical Properties: Y_3_, Y_4,_ and Y_5_

The porosity *ε_%_* (*Y_3_*) is the third independent geometrical descriptor of these scaffolds and conveys relevant information about fiber density and scaffold function. It is in fact linked to the efficient transport of nutrients, gases, and waste products [7,23,41,44]. Because porosity is crucial to cell migration and engraftment, it can be the controlling factor of scaffold performance and adversely affect cell viability. Scaffold porosity varies considerably in Table 4, in the range of 65–90%. The most porous scaffold is T2 (90%), followed by T5 (86%). Minimum porosity was obtained for T4 (65%). It is known that porosity is closely related to cell mobility and promotes cell adhesion, in conjunction with the high surface area density of electrospun scaffolds [7,44,45]. Bioactive scaffolds in the literature often have a porosity greater than 80%, which is a requirement achievable by properly controlling the FR and V in the case presented in this study.

The next scaffold descriptor is wettability vs. water, quantified using the contact angle (CA), which affects the way a biomaterial surface interacts with the biological environment [46]. Thus, a superficial characterization of the contact angle (*Y*_4_) provides a potentially important descriptor for understanding the observed biological effects. The average CA for each treatment is between 111.52° (T4) and 127.44° (T2), confirming a rather hydrophobic behavior of all scaffolds, typical of PCL [47]. As expected, the lower values of CA in Table 4 correspond to higher values of fiber diameters [39]. Noteworthy, this hydrophobicity does not hinder cell adhesion since scaffolds are conditioned before biological testing as described in Section 2.7. 

The last descriptor (*Y_5_*) relates to the elastic response of the polymeric scaffold expressed in terms of Young’s modulus *E*. The mechanical (elastic) properties are instrumental in fine-tuning the scaffold function and driving cell differentiation in tissue engineering [48,49,50,51,52,53]. Young’s modulus varies greatly, spanning over one order of magnitude range, largely. The highest *E* value is obtained for T4 (39 MPa), whereas T2 showed the lowest value (3 MPa). Expectedly, electrospun materials made of thicker fibers tend to be stiffer, with higher yield stresses. Instead, no obvious correlation between *E* and scaffold porosity can be identified at a glance from raw data. 

#### 3.1.3. Biological Evaluation: Y_6_ 

**Figure 5:**
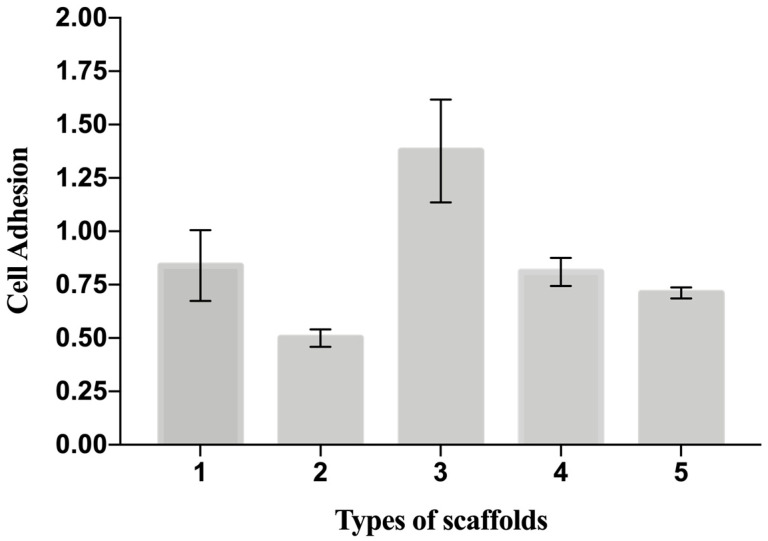
Cell adhesion on the scaffolds measured as ratio number of cells attaching to the scaffold to the number of cells attaching to the well. Data are expressed as the mean ± SD (*p*-values < 0.05 is the significance interval).

**Figure 6:**
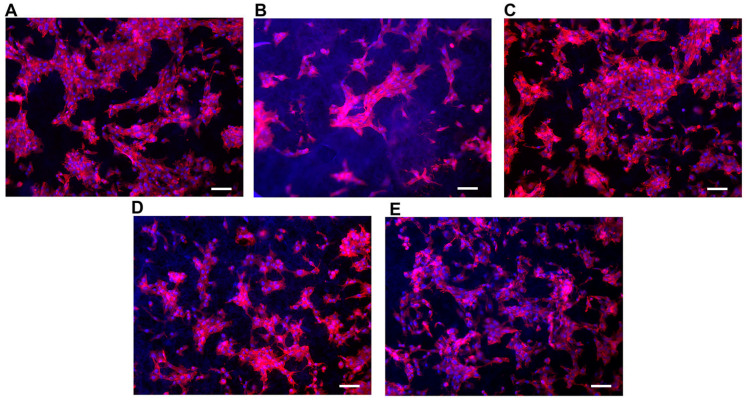
Representative images of C2C12 cells on the different types of scaffolds showing F-actin (red) into cytoplasm and nuclei stained with DAPI (Blue). Scale bars = 30 μm for (**A**–**E**).

The cellular adhesion in Table 4 is a useful indicator of cell vitality and the toxicity of a biomaterial. As such, in our study, a cell adhesion assay (*Y*_6_) represents the basic way to probe and compare the biocompatibility of the scaffolds. The adhesion of C2C12 cells on different PCL scaffolds (normalized by cell number adhering on the bottom of the well area) at 48 h after seeding is shown in Figure 5. The results show that cell adhesion was maximum on the T3 scaffold. Moreover, the values of cells adhesion for T1-T3-T4 were higher than for the control (polystyrene plate). Figure 6 provides some DAPI/F actin-stained cells over the scaffold, demonstrating the good cell viability of C2C12 cells on all these PCL scaffolds.

### 3.2. ANOVA Models for Ys

The results from fitting a full 2^2^ factorial model to each *Y*_1–6_ are reported in Table 5, showing the coefficients of Equation (4) and the corresponding *p*-values to decide whether that term is significant or not based on the selected significance level of 10%. The *R*^2^ and *R*^2^-*adj* are displayed at the bottom for each output *Y*, with *R*^2^ values in the 67–99% range, indicating “good” to “very good” agreement with the experimental data. *R*^2^-*adj* values just moderately decrease and are acceptable, with the *Y*_5_ model exhibiting the most modest performance (*R*^2^ = 67.50%, *R*^2^-*adj* = 56.63%). The reported *R*^2^ values are obtained by removing terms from Equation (4) in favor of a simpler model (also called a non-hierarchical model) and retaining only the most relevant effects and interactions using a “step-wise backward” regression approach [35]. 

**Figure 7:**
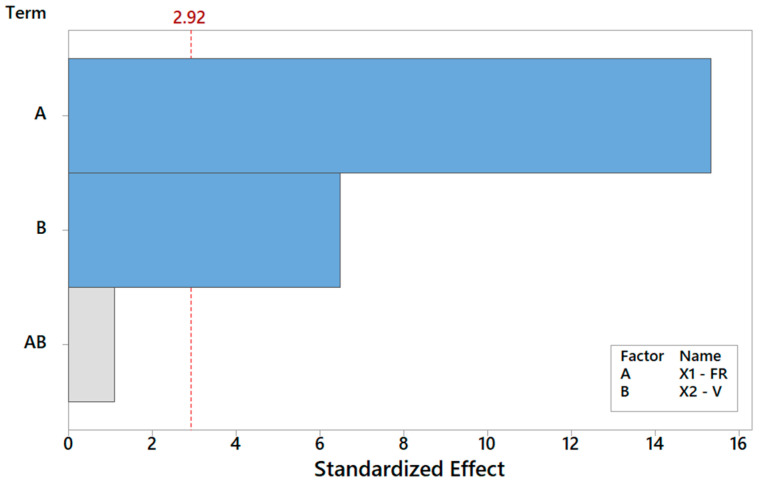
Pareto plot of standardized effects for Y_1_, rendering a visual ranking of their role on the FD. A gray bar represents a term not in the model.

Let us discuss the procedure to select a reduced model for *Y*_1_. As mentioned above, one major benefit of the DOE factorial approach is the possibility to quickly refine the analysis and identify the simplest model by removing non-significant terms. By proceeding step wise from a full model, the backward elimination algorithm starts with all predictors in the model and removes the least significant variable for each step one at a time. The MINITAB algorithm stops when all variables in the model have *p*-values that are less than or equal to the specific significance value, i.e., 10% in our case. Table 6 provides the *Y*_1_ model generated by “dropping” all terms that exhibit a *p*-value > 0.1, where two terms are found to be significant. Note that the coefficients of the “kept” terms do not need to be re-estimated because of the orthogonality property of the DOE. The relevant terms can be ranked by importance either by their (decreasing) *p*-values or, more intuitively, via their (increasing) t-values [34,35,36]. For coded models, the absolute value of each t-value conveys a standardized measure of the associated effect/interaction and can be arranged in a Pareto histogram as portrayed in Figure 7, which offers an overall pictorial perspective that is more convenient to comprehend and analyze than Table 5. The model explains 99.28% of the variability in *Y*_1_ and can predict the mean FD to within ±0.348 μm (±2·MSE), corresponding to a 90% confidence level. In terms of the t-value, any effect in Figure 7 that exceeds the reference line for t_0_ = 2.92 is significant at a 10% significance level, consistent with Table 6. The single most important effect on *Y*_1_ is the FR (ref. A = X_1_), followed by V (ref. B = X_1_). The FR and V are the real controlling variables of the FD, whereas the interaction term between the FR and V is negligible, as correctly concluded in Section 3.1.1.

**Figure 8:**
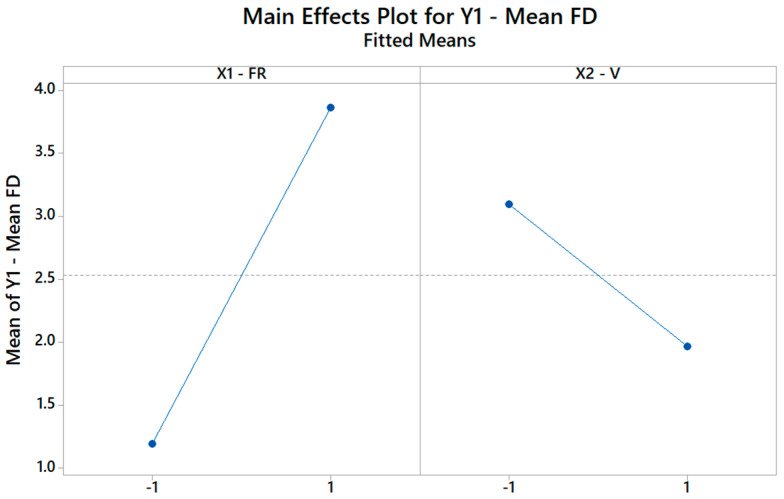
The main effects plot for the FD plot showing how the model explains the change in mean FD based on the change of the FR and V levels.

Figure 8 illustrates how the FD changes when the FR changes from its low level (−1) to its high level (+1), assuming the other factor, i.e., V is kept constant, and vice versa. Remarkably, since C_2_ is negative, increasing the V (X_2_) does increase the FD. Hence, through the DOE, it is possible to not only identify but also quantify the role of each input parameter on the selected output properties. 

**Figure 9:**
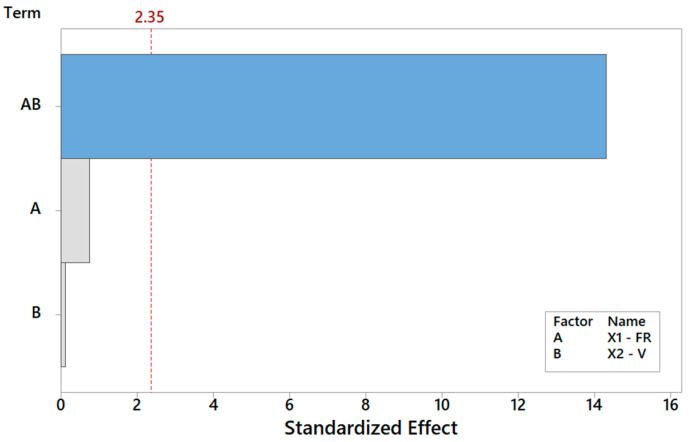
Pareto plot of standardized effects for Y_2_, rendering a visual ranking of their role on the spread of the FD. A gray bar represents a term not in the model.

**Figure 10:**
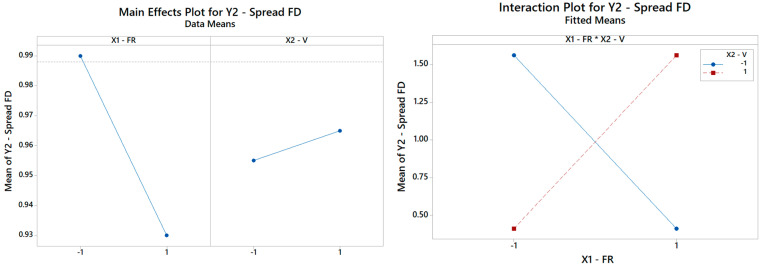
(**Left**) The main effects plot and (**right**) the interaction plot for the spread of FD (only the interaction term is significant and retained in the final model as per Table 7).

The rationale holds identical for *Y*_2_, *Y*_3,_ *Y*_4_, *Y*_5,_ and *Y*_6_. By repeating the same search procedure for *Y*_2_, the reduced model in Table 5 is achieved, where the interaction between the FR and V turns out to be the most and only significant term affecting the spread of the fiber distribution (Table 7). The Pareto ranking for *Y*_2_ is on display in Figure 9. Thus, while neither main effect V nor FR is crucial per se in the determination of the FD RMS, each of them can completely revert the impact of the other main effect, as highlighted in Figure 10. The primary role of the interactions has received little attention in the literature, but it is an elusive aspect that cannot be pinpointed without a systematic DOE study. Here, Table 7 indicates that the fitted model is appropriate (with a high coefficient of determination of about 99.99%) and can estimate (interpolate) data trends very accurately for engineering purposes. 

For *Y*_3_, *Y*_4_, *Y*_5_, the analysis yields the results in Table 5 (additional tables and figures are reported in the Supplementary Material). Briefly, (i) in the case of porosity *Y*_3_, the scenario is analogous to *Y*_2_ since the interaction FR·V is again most relevant; and (ii) for the contact angle *Y*_4_ and for the mechanical property *Y*_5_, the main effect FR is dominant, with no significant interaction. For *Y*_5_, the relatively low *R*^2^ (67.50%) may suggest running a refined factorial design and checking for lack of fit due to curvature in our data, for example implementing a response surface DOE design such as a central composite design [35].

**Figure 11:**
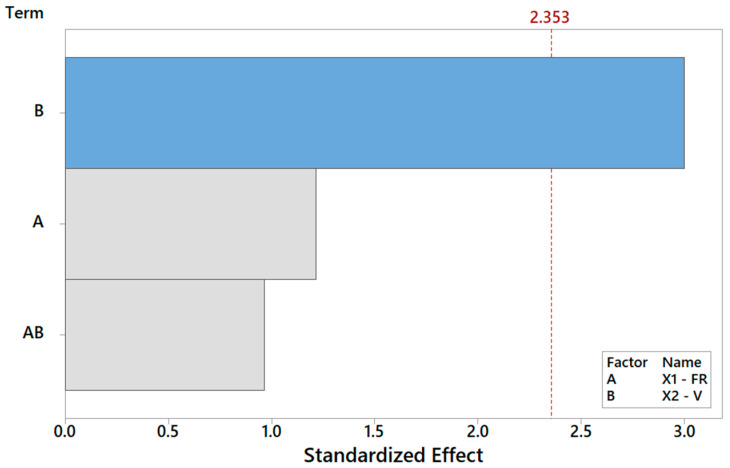
Pareto plot of standardized effects for Y_6_ from Table 8, rendering a visual ranking of their role on cell adhesion. A gray bar represents a term not in the model.

**Figure 12:**
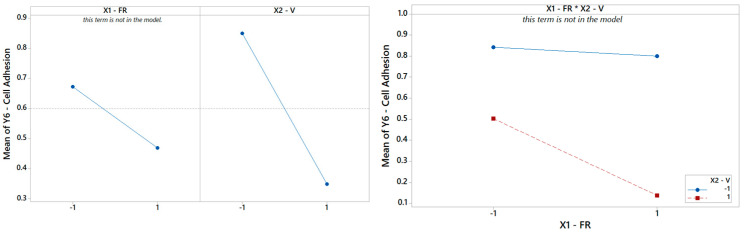
(**Left**) The main effects plot and (**right**) the interaction plot for cell adhesion.

Finally, focusing on the last output parameter in our analysis *Y*_6_, the biocompatibility of the PCL scaffolds is captured using a reduced model where only the main effect V is significant, according to Table 8. The Pareto ranking and the factor plots for *Y*_6_ are displayed in Figure 11 and Figure 12. The modest *R*^2^ (75%) is an acceptable result for engineering purposes, also considering the extreme simplicity of 1-term model, but it also means that more information (e.g., more observations, more regressors, or higher terms) could be included in future analysis to substantially improve the statistical fitting. 

### 3.3. Implication of DOE for Design of Multilayer Electrospun Scaffold: T-MIX Example

In order to reproduce the typical cell environment, sometimes a scaffold has to be designed with multiple length scales across its thickness, with multiple zones or with compartments optimized for specific cell types. The information accrued using the modeling analysis proposed above may have a profound impact in this respect since it can be used to aid in the design and manufacturing of such complex scaffolds using electrospinning. For illustrative purpose, let us suppose we create a “derived two-layer scaffold”, labelled as scaffold T-MIX in Table 9 and obtained from parent scaffolds T1 and T3. The latter two are here chosen arbitrarily, although they correspond to the DOE treatments exhibiting the best biological performances while being quite different from the microstructural point of view, making for an interesting case study aiming at a compound microstructure with high morphological and mechanical gradients. 

**Figure 13:**
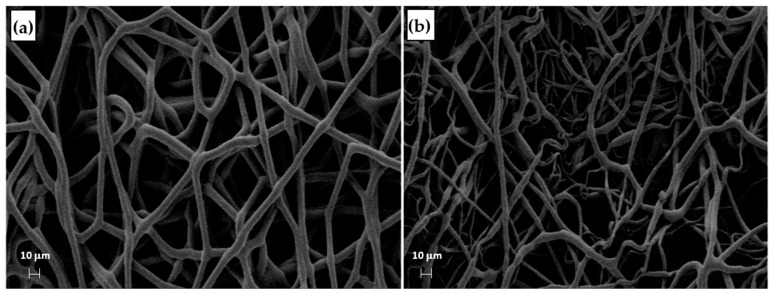
SEM micrographs of the bottom and top of the by-layer scaffold T-MIX: (**a**) side T3 and (**b**) side T1.

The process parameters and the Ys of the T-MIX vs. parent scaffolds T1 and T3 are summarized in Table 9, indicating that the T-MIX scaffold indeed inherited some of their key properties. The mean FD and RMS FD values of each “layer T1” and “layer T3” of the T-MIX are remarkably similar to the respective values of the scaffolds T1 and T3 (Figure 13), confirming that T-MIX is endowed with marked through-thickness gradients. The other reported metrics in Table 9 (i.e., the contact angle and the cell adhesion) are on average intermediate between T1 and T3. While the T-MIX scaffold retains a good biological response in spite of the microstructural (and thus mechanical) gradients across the two layers, the possibility to create layer-by-layer scaffolds with the controlled fiber distribution in each layer is perhaps the most telling result from this example here, paving the way to the deployment of the rationale design of TE scaffolds using electrospinning aided by the DOE.

### 3.4. Additional Remarks about ANOVA and Correlations between Ys

**Figure 14:**
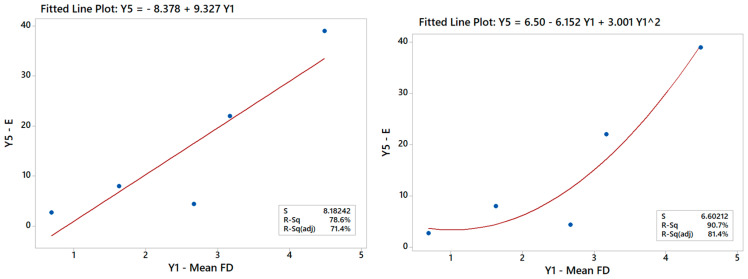
Linear model of Y_5_ vs. Y_1_ indicating a good correlation between these output variables.

Generally speaking, establishing correlations between the Ys is useful for engineering purposes and represents a valid challenge for future studies to improve the DOE results, as already pointed out in a previous paper [24]. The DOE analysis of the Y–X relationships can be augmented indeed with additional regression studies exploring the Y–Y relationships between outputs in the given multi-response dataset (ref. Table 5). For example, it is possible to run an ANOVA to correlate Y_5_ with Y_1_, especially since scaffolds with larger FDs were discussed to be generally stiffer in Section 3.1.2. The linear model (*R*^2^ > 78.6%) in Figure 14 in fact confirms this trend but also points out that the data may suggest a curvature effect. A quadratic model actually improves the data fit considerably (*R*^2^ > 90%) and renders a better model for predicting Young’s modulus from the mean FD for the scaffolds and vice versa. Such a model hence provides an alternative option that can be used either synergistically or in place of the DOE models, depending on the design or manufacturing task at hand.

Yet, the pursuit of Y–Y models is not a trivial task. In this regard, for example, in this specific study, none of the physical properties (Y_1–5_) appear to correlate easily with the biological response (Y_6_), and no satisfactory reduced model can be identified for Y_6_ regressed on Y_1–5_. Similar to the DOE, larger datasets, more regressors, and—if necessary—higher-order linear models need to be accounted for to improve the likelihood of a satisfactory regression analysis. 

## 4. Conclusions

The results of this study support that the implementation of statistical process mapping of electrospinning is possible and that the resulting statistical models can be useful to develop better TE scaffolds.

While expanding and reinforcing the conclusions from an earlier study [23,24], the DOE approach described herein represents a flexible and modular design platform that can be expanded or reduced in scope and size, depending on the complexity and design goals case-by-case. The methodology is quite powerful considering that the case discussed in this paper was elaborated on a rather limited dataset of five unreplicated treatments only, yet yielding statistical correlations and regression models of high significance. The data obtained from this study encourage the adoption of the DOE strategy in the design and manufacturing of TE scaffolds using electrospinning. As far as electrospinning is concerned, the findings reveal that the flow rate (FR) is often the most important process parameter (main effect) for several scaffold properties but that the importance of interaction effects can be even larger than any single main effect, as confirmed by the Pareto plots (Figure 9). Therefore, a DOE-like method is a proper way to characterize and understand an electrospinning process at hand and the resulting electrospun scaffolds, thus enabling accurate interpolation within the design domain and the implementation of a material-by-design approach to manufacturing.

Additionally, once understood how the process parameters influence the scaffold properties, it appears possible to pursue multiscale layer-by-layer scaffolds with desired microstructural gradient, useful to reproduce a complex ECM environment and direct cellular response for regenerative purposes. In this regard, the rapid increase in the size of DOE datasets with a number of Xs and Ys may pose some constraints, which, in our view, can be effectively addressed either using reduced designs (e.g., “fractional” designs, “subsets analysis”) or using “artificial intelligence” tools. 

Finally, the far-reaching impact of our findings goes beyond electrospinning since the proposed approach per se is applicable to scaffolds produced using any other advanced manufacturing techniques (e.g., 3D printing or chemical self-assembling). 

## Figures and Tables

**Table 1:** The *Xs* set—material and processing parameters in electrospinning (adapted from [23]).

**Solution Properties**
Viscosity Polymer concentration Molecular weight of polymer Electrical conductivity Elasticity Surface tension
**Processing parameters**
Applied voltage
Distance from needle tip to collector
Feeding rate
Needle diameter
Collector composition and geometry
**Ambient parameters (which can be “processing parameters” in equipment with an environmental control unit”)**
Temperature Humidity Atmosphere pressure

**Table 2:** Inputs (*Xs*)—the two selected materials and processing parameters in this investigation.

	Parameter	Unit	LOW Level (−1)	HIGH Level (+1)	Mean Level (0)
*X*_1_	Voltage	kV	26	32	29
*X*_2_	Flow Rate	mL/h	4	6	5

**Table 3:** Outputs (*Ys*)—selected scaffold properties/functions examined in this investigation.

	Parameter	Unit	Label
*Y*_1_	Mean FD	μm	FD
*Y*_2_	Spread FD	μm	SD
*Y*_3_	Porosity	%	*ε*%
*Y*_4_	Contact Angle	°	CA
*Y*_5_	Young’s modulus	MPa	*E*
*Y*_6_	Cell Adhesion	n° cells/mL	-

**Table 4:** Summary experimental matrix with input and output data from the DOE analysis.

Treatments	Inputs (X_s_) *			Outputs (Y_s_)					
	Flow Rate	Voltage		Mean FD (µm)	Spread FD (µm)	ε (%)	CA (°)	E (MPa)	Cell Adhesion
	X_1_	X_2_		Y_1_	Y_2_	Y_3_	Y_4_	Y_5_	Y_6_
T1	−1	−1		1.63	1.56	70	123.98	8.0	0.842
T2	−1	1		0.69	0.42	90	127.44	2.7	0.502
T3	1	1		3.17	1.51	65	114.72	22.0	1.379
T4	1	−1		4.49	0.35	86	111.52	39.0	0.800
T5	0	0		2.67	1.10	75	117.25	4.4	0.714

* coded units.

**Table 5:** Fitted coefficients for the factorial model in Equation (4) fitted in coded units for Y_1–6_ along with *p*-values * per each term and the global *R*^2^. *p*-values smaller than 0.001 are displayed as “<0.001”.

		Y_1_ Mean FD (μm)		Y_2_ Spread FD (μm)		Y_3_ ε (%)		Y_4_ CA (°)		Y_5_ E (MPa)		Y_6_ Cell Adhesion	
Model Coefficients	Main Effect/Interaction	C_ijk_	*p*	C_ijk_	*p*	C_ijk_	*p*	C_ijk_	*p*	C_ijk_	*p*	C_ijk_	*p*
(C_0_) *		2.53	<0.001	0.99	<0.001	77.20	<0.001	118.98	<0.001	15.22	0.043	0.07	0.004
C_1_	X_1_	1.34	0.004	-	-	-	-	-6.30	0.011	12.57	0.088	-	-
C_2_	X_2_	−0.56	0.023	-	-	-	-	-	-	-	-	−0.25	0.058
C_12_	X_1_·X_2_	-	-	0.58	0.001	−10.25	0.006	-	-	-	-	-	-
*R* ^2^		99.28%		98.56%		94.06%		91.43%		67.48%		75.01%	
*R* ^2^ *(adj)*		98.56%		98.07%		92.08%		88.57%		56.63%		66.68%	

* C_0_ is a fitting constant not corresponding to any X or combination of Xs.

**Table 6:** Model in coded units for output variable *Y_1_* obtained by dropping terms with *p*-value > 0.1.

Y_1_—Mean FD (µm)						
C_ij_	Main Effect/ Interaction	Model	Standardized Effect (t-Value)	*p*	Keep/Drop	Rank by Significance
(C_0_)		2.53	32.50	<0.001	(Keep)	(0)
C_1_	X_1_	1.34	15.34	0.004	Keep	1
C_2_	X_2_	-0.56	-6.49	0.023	Keep	2
C_12_	X_1_·X_2_	-	-	-	Drop	-
*R* ^2^		99.28%				
Variance		0.174				

**Table 7:** Model in coded units for output variable *Y_2_* obtained by dropping terms with *p*-value > 0.1.

Y_2_—RMS FD (µm)						
C_ij_	Main Effect/ Interaction	Model	Standardized Effect (t-Value)	*p*	Keep/Drop	Rank by Significance
(C_0_)		0.99	27.49	<0.001	(Keep)	
C_1_	X_1_	-	-	-	Drop	-
C_2_	X_2_	-	-	-	Drop	-
C_12_	X_1_·X_2_	0.14	14.31	0.001	Keep	1
*R* ^2^		98.56%				
Variance		0.080				

**Table 8:** Model in coded units for output variable *Y_6_* obtained by dropping terms with *p*-value > 0.1.

Y_6_—Cell Adhesion						
C_ij_	Main Effect/ Interaction	Model	Standardized Effect (t-Value)	*p*	Keep/Drop	Rank by Significance
(C_0_)		0.60	8.02	0.004	(Keep)	
C_1_	X_1_	-	-	-	Drop	-
C_2_	X_2_	−0.25	−3.00	0.058	Keep	1
C_12_	X_1_·X_2_	-	-	-	Drop	-
*R* ^2^		75.01%				
Variance		0.167				

**Table 9:** Input and output data of the samples T-MIX, T1, and T3, to directly compare the by-layer system vs the parent scaffolds. All T1, T3, and T-MIX were electrospun for 10 min with “layer T1” and “layer T3”.

Treatment		Inputs (X_S_)			Outputs (Y_S_)			
		FR	V		Mean FD (µm)	Spread SD (µm)	CA (°)	Cell Adhesion
T-MIX	layer T1	−1	−1		1.98	1.89	116.00 *	1.471 *
	layer T3	1	1		4.23	1.54		
T1		−1	−1		1.63	1.56	123.98	0.400
T3		1	1		3.17	1.51	114.72	1.379

* effective properties of the T-MIX scaffold.

## Data Availability

Not applicable.

## Supplementary Materials

The following supporting information can be downloaded at: https://www.mdpi.com/article/10.3390/ma16041539/s1, Figures S1–S3: Pareto and interaction plots, respectively, for Y_3_, Y_4_, and Y_5_. Tables S1–S3: ANOVA table, respectively, for Y_3_, Y_4_, and Y_5_. Figure S4: The provided stress–strain curves.
