# Supplementary material for: Towards a Material-by-Design Approach to Electrospun Scaffolds for Tissue Engineering Based on Statistical Design of Experiments (DOE)
Source: Materials (Basel). 2023 Feb 12;16(4):1539. doi: 10.3390/ma16041539 (PMC9961090; doi:10.3390/ma16041539)
Supplement: Supplementary file 1 [file materials-16-01539-s001.zip › materials-2162178-supplementary.pdf]

materials

MDPI

Article

Towards a material-by-design approach to electrospun scaffolds for tissue engineering based on statistical design of experiments (DOE)

Felicia Carotenuto [1,2,†], Noemi Fiaschini [3,†], Paolo Di Nardo [1,2] and Antonio Rinaldi [4,*]

1 Dipartimento di Scienze Cliniche e Medicina Traslazionale, Università degli Studi di Roma "Tor Vergata", Via Montpellier 1, 00133 Rome, Italy;
2 CIMER-Centro di Ricerca Interdipartimentale di Medicina Rigenerativa, Università degli Studi di Roma "Tor Vergata", Via Montpellier 1, 00133 Rome, Italy
3 NANOFABER S.r.l., Via Anguillarese 301, 00123 Rome, Italy
4 SSPT-PROMAS-MATPRO Laboratory, ENEA—Italian National Agency for New Technologies, Energy and Sustainable Economic Development, Via Anguillarese 301, 00123 Rome, Italy;
* Correspondence: antonio.rinaldi@enea.it (A.R.)
† These authors contributed equally to this work.

SUPPLEMENTARY MATERIAL

- **ADDITIONAL DATA FROM ANOVA**

Table S1: The model in coded units for output variable Y_3 obtained by dropping all terms with *p-value* > 0.1 (i.e. at a significance level of 0.1)

$Y_3 - \varepsilon$ (%)

C_{ij}	Main Effect/ Interaction	Model	Standardized effect (t-value)	*p*	Keep/ Drop	Rank by Significance
(C_0)		77.2	58.03	<0.001	(Keep)	
C_1	X_1	-	-	-	Drop	-
C_2	X_2	-	-	-	Drop	-
C_{12}	$X_1 \cdot X_2$	-10.25	-6.89	0.006	Keep	1
R^2		94.06%				
Variance		2.97				

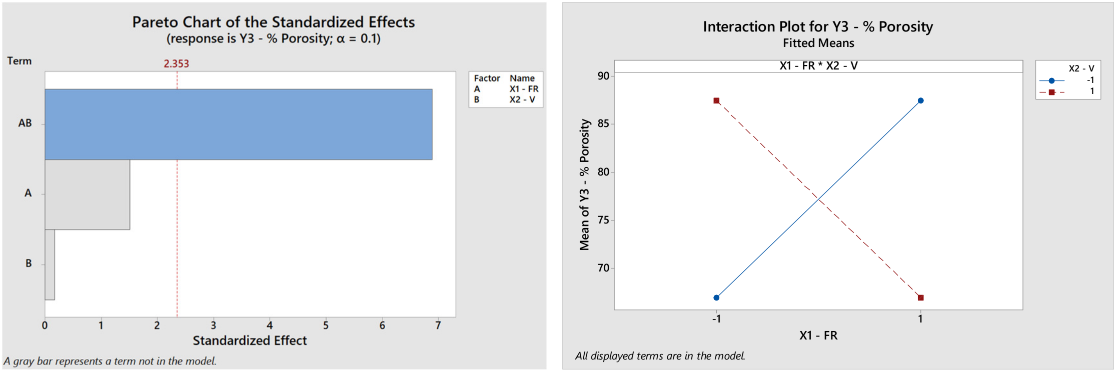

Figure S1: Pareto plot of standardized effects for Y_3 from Table S1, rendering a visual ranking of their role on the porosity and interaction plot for ε% (a gray bar represents a term not in the model).

Table S2: The model in coded units for output variable Y_4 obtained by dropping all terms with *p-value* > 0.1 (i.e. at a significance level of 0.1)

**Y_4 – CA (°)**						
C_{ij}	Main Effect/ Interaction	Model	Standardized effect (t-value)	*p*	Keep/ Drop	Rank by Significance
(C_0)		118.98	1825.96	<0.001	(Keep)	
C_1	X_1	-6.30	-5.66	0.011	Keep	1
C_2	X_2	-	-	-	Drop	
C_{12}	$X_1 \cdot X_2$	-	-	-	Drop	-
R^2		91.43%				
Variance		2.225				

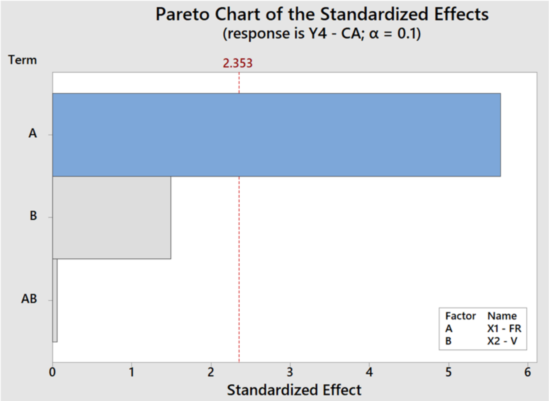

Figure S2: Pareto plot of standardized effects for Y_4 from Table S2, rendering a visual ranking of their role on the porosity for CA (a gray bar represents a term not in the model).

Table S3: The model in coded units for output variable Y_5 obtained by dropping all terms with *p-value* > 0.1 (i.e. at a significance level of 0.1)

Y_5 – E (MPa)						
C_{ij}	Main Effect/ Interaction	Model	Standardized effect (t-value)	*p*	Keep/ Drop	Rank by Significance
(C_0)		15.24	3.38	0.043	(Keep)	
C_1	X_1	12.56	2.5	0.088	Keep	1
C_2	X_2	-	-	-	Drop	-
C_{12}	$X_1 \cdot X_2$	-	-	-	Drop	-
R^2		67.50%				
Variance		10.066				

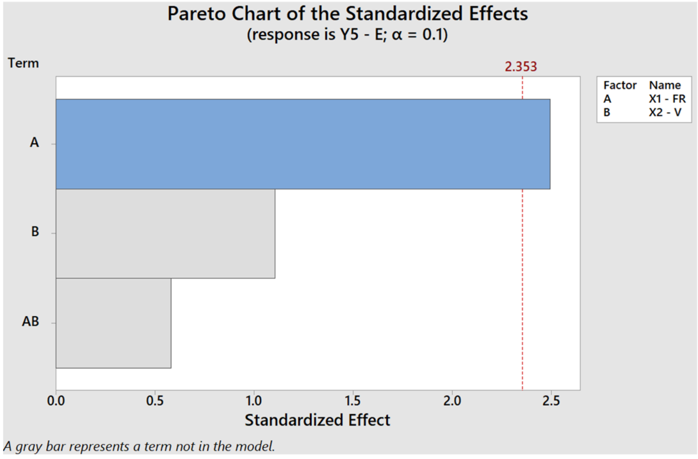

Figure S3: Pareto plot of standardized effects for Y_5 from Table S3, rendering a visual ranking of their role on the Young’s modulus.

- **ADDITIONAL DATA FROM MECHANICAL TESTING**

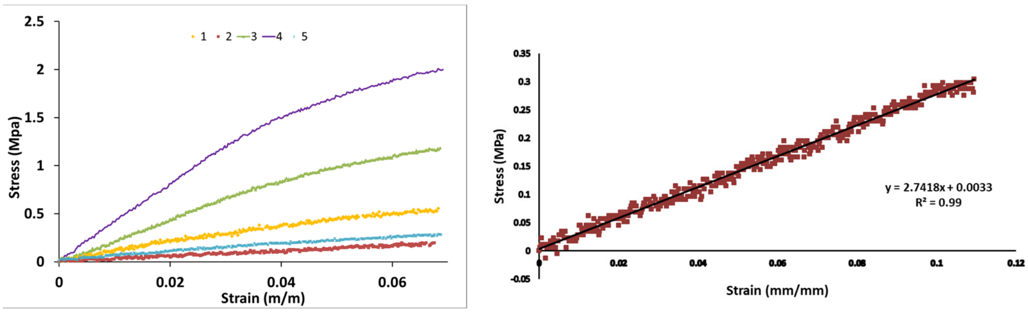

Figure S4: Stress vs. strain responses of the five scaffolds and example of linear region used to estimate the Young's modulus for T2.
